# *N*′-(5-Bromo-1*H*-indol-3-ylmethyl­idene)-3,4,5-trihydroxy­benzohydrazide

**DOI:** 10.1107/S1600536808031991

**Published:** 2008-10-11

**Authors:** Hamid Khaledi, Hapipah Mohd Ali, Seik Weng Ng

**Affiliations:** a Department of Chemistry, University of Malaya, 50603 Kuala Lumpur, Malaysia

## Abstract

The two aromatic parts of the title mol­ecule, C_16_H_12_BrN_3_O_4_, are connected through a conjugated –CH=N—NH—C(O)– fragment to furnish an almost planar mol­ecule. Adjacent mol­ecules are linked by N—H⋯O and O—H⋯O hydrogen bonds into a three-dimensional network. An intramolecular O—H⋯O link also occurs.

## Related literature

For other Schiff bases derived by condensing 5-bromo-1*H*-indole-3-carbaldehyde with aroylhydrazines, see: Ali *et al.* (2005*a* ▶,*b* ▶,*c* ▶).
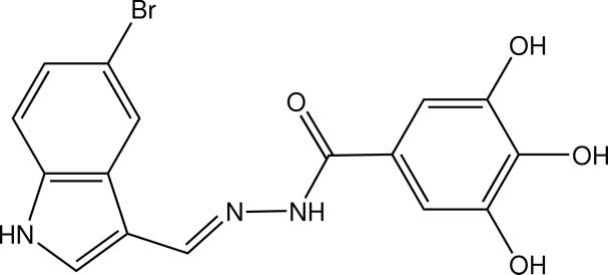


## Experimental

### 

#### Crystal data

C_16_H_12_BrN_3_O_4_
*M* *_r_* = 390.20
Monoclinic, 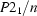

*a* = 9.6454 (2) Å
*b* = 14.9694 (4) Å
*c* = 10.3845 (2) Å
β = 97.390 (1)°
*V* = 1486.92 (6) Å^3^
*Z* = 4
Mo *K*α radiation
μ = 2.79 mm^−1^
*T* = 100 (2) K
0.40 × 0.25 × 0.10 mm

#### Data collection

Bruker SMART APEX diffractometer
Absorption correction: multi-scan (*SADABS*; Sheldrick, 1996 ▶) *T*_min_ = 0.401, *T*_max_ = 0.768
10182 measured reflections
3403 independent reflections
2786 reflections with *I* > 2σ(*I*)
*R*_int_ = 0.024

#### Refinement

*R*[*F* ^2^ > 2σ(*F* ^2^)] = 0.029
*wR*(*F* ^2^) = 0.078
*S* = 1.02
3403 reflections
220 parameters
H-atom parameters constrained
Δρ_max_ = 0.62 e Å^−3^
Δρ_min_ = −0.36 e Å^−3^

### 

Data collection: *APEX2* (Bruker, 2007 ▶); cell refinement: *SAINT* (Bruker, 2007 ▶); data reduction: *SAINT*; program(s) used to solve structure: *SHELXS97* (Sheldrick, 2008 ▶); program(s) used to refine structure: *SHELXL97* (Sheldrick, 2008 ▶); molecular graphics: *X-SEED* (Barbour, 2001 ▶); software used to prepare material for publication: *publCIF* (Westrip, 2008 ▶).

## Supplementary Material

Crystal structure: contains datablocks global, I. DOI: 10.1107/S1600536808031991/tk2310sup1.cif

Structure factors: contains datablocks I. DOI: 10.1107/S1600536808031991/tk2310Isup2.hkl

Additional supplementary materials:  crystallographic information; 3D view; checkCIF report

## Figures and Tables

**Table 1:** Hydrogen-bond geometry (Å, °)

*D*—H⋯*A*	*D*—H	H⋯*A*	*D*⋯*A*	*D*—H⋯*A*
O2—H2O⋯O3	0.84	2.21	2.681 (2)	116
O3—H3O⋯O1^i^	0.84	1.76	2.595 (2)	173
O4—H4O⋯N2^i^	0.84	2.02	2.778 (2)	150
N1—H1N⋯O2^ii^	0.88	2.26	3.111 (2)	163
N3—H3N⋯O4^iii^	0.88	2.11	2.932 (2)	154

Symmetry codes: (i) 
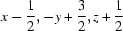
; (ii) 
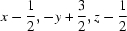
; (iii) 
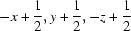
.

**Fig. 1.**
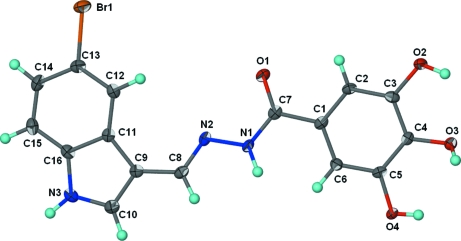
Thermal ellipsoid plot (Barbour, 2001) of (I) at the 70% probability level. Hydrogen atoms are drawn as spheres of arbitrary radius.

## supplementary crystallographic information

### Comment

The molecule of (I), Fig. 1, is almost planar with the aromatic groups connected via a conjugated –CH═N–NH–C(O)– fragment. Molecules are connected into a 3-D network via N—H···O and O—H···O hydrogen bonds, Table 1.

### Experimental

5-Bromoindole-3-carbaldehyde (0.34 g, 1.5 mmol) and 3,4,5-trihydroxybenzoylhydrazine (0.27 g, 1.5 mmol) were heated in ethanol (20 ml) for 3 h.  About 1 ml of acetic acid also added. The solution was set aside for the growth of crystals.

### Refinement

Hydrogen atoms were placed at calculated positions (C—H 0.95, N—H 0.88 and O—H 0.84 Å) and were treated as riding on their parent atoms, with *U*(H) set to 1.2–1.5 times *U*_eq_(C,N,O). For the hydroxy groups, an *sp*^2^ type of hybridization was assumed.

### Crystal data

**Table:**

C_16_H_12_BrN_3_O_4_	*F*(000) = 784
*M**_r_* = 390.20	*D*_x_ = 1.743 Mg m^−^^3^
Monoclinic, *P*2_1_/*n*	Mo *K*α radiation, λ = 0.71073 Å
Hall symbol: -P 2yn	Cell parameters from 3960 reflections
*a* = 9.6454 (2) Å	θ = 2.4–28.2°
*b* = 14.9694 (4) Å	µ = 2.79 mm^−^^1^
*c* = 10.3845 (2) Å	*T* = 100 K
β = 97.390 (1)°	Block, orange
*V* = 1486.92 (6) Å^3^	0.40 × 0.25 × 0.10 mm
*Z* = 4	

### Data collection

**Table:**

Bruker SMART APEX diffractometer	3403 independent reflections
Radiation source: fine-focus sealed tube	2786 reflections with *I* > 2σ(*I*)
graphite	*R*_int_ = 0.024
ω scans	θ_max_ = 27.5°, θ_min_ = 2.4°
Absorption correction: multi-scan (SADABS; Sheldrick, 1996)	*h* = −12→11
*T*_min_ = 0.401, *T*_max_ = 0.768	*k* = −19→19
10182 measured reflections	*l* = −13→13

### Refinement

**Table:**

Refinement on *F*^2^	Primary atom site location: structure-invariant direct methods
Least-squares matrix: full	Secondary atom site location: difference Fourier map
*R*[*F*^2^ > 2σ(*F*^2^)] = 0.029	Hydrogen site location: inferred from neighbouring sites
*wR*(*F*^2^) = 0.078	H-atom parameters constrained
*S* = 1.02	*w* = 1/[σ^2^(*F*_o_^2^) + (0.0419*P*)^2^ + 0.9663*P*] where *P* = (*F*_o_^2^ + 2*F*_c_^2^)/3
3403 reflections	(Δ/σ)_max_ = 0.003
220 parameters	Δρ_max_ = 0.62 e Å^−^^3^
0 restraints	Δρ_min_ = −0.36 e Å^−^^3^

### Special details

**Table:**

Geometry. All e.s.d.'s (except the e.s.d. in the dihedral angle between two l.s. planes) are estimated using the full covariance matrix. The cell e.s.d.'s are taken into account individually in the estimation of e.s.d.'s in distances, angles and torsion angles; correlations between e.s.d.'s in cell parameters are only used when they are defined by crystal symmetry. An approximate (isotropic) treatment of cell e.s.d.'s is used for estimating e.s.d.'s involving l.s. planes.

### Fractional atomic coordinates and isotropic or equivalent isotropic displacement parameters (Å^2^)

**Table:**

	*x*	*y*	*z*	*U*_iso_*/*U*_eq_	
Br1	1.00401 (2)	1.090710 (16)	0.25205 (2)	0.02243 (8)	
O1	0.69032 (15)	0.83598 (10)	0.56939 (13)	0.0157 (3)	
O2	0.64359 (15)	0.62930 (10)	0.94165 (13)	0.0163 (3)	
H2O	0.5987	0.5999	0.9914	0.024*	
O3	0.37031 (15)	0.59355 (9)	0.93388 (13)	0.0133 (3)	
H3O	0.3085	0.6178	0.9721	0.020*	
O4	0.17544 (15)	0.67374 (10)	0.73325 (13)	0.0141 (3)	
H4O	0.1569	0.6421	0.7956	0.021*	
N1	0.46882 (18)	0.86863 (11)	0.48158 (15)	0.0134 (3)	
H1N	0.3788	0.8619	0.4852	0.016*	
N2	0.51457 (18)	0.92346 (11)	0.38712 (16)	0.0129 (3)	
N3	0.40143 (19)	1.10565 (11)	0.03097 (16)	0.0151 (4)	
H3N	0.3557	1.1326	−0.0369	0.018*	
C1	0.5056 (2)	0.76400 (13)	0.66032 (18)	0.0123 (4)	
C2	0.6012 (2)	0.72451 (13)	0.75620 (18)	0.0129 (4)	
H2	0.6984	0.7361	0.7598	0.016*	
C3	0.5519 (2)	0.66829 (13)	0.84567 (18)	0.0128 (4)	
C4	0.4097 (2)	0.65084 (13)	0.84261 (18)	0.0116 (4)	
C5	0.3160 (2)	0.68919 (13)	0.74520 (18)	0.0122 (4)	
C6	0.3639 (2)	0.74523 (13)	0.65414 (18)	0.0124 (4)	
H6	0.2996	0.7709	0.5873	0.015*	
C7	0.5628 (2)	0.82603 (13)	0.56719 (18)	0.0128 (4)	
C8	0.4168 (2)	0.96589 (13)	0.31563 (19)	0.0133 (4)	
H8	0.3229	0.9587	0.3324	0.016*	
C9	0.4446 (2)	1.02350 (13)	0.21187 (19)	0.0125 (4)	
C10	0.3424 (2)	1.05580 (14)	0.11812 (19)	0.0150 (4)	
H10	0.2451	1.0447	0.1152	0.018*	
C11	0.5760 (2)	1.05663 (13)	0.17868 (18)	0.0121 (4)	
C12	0.7151 (2)	1.05011 (13)	0.23622 (18)	0.0135 (4)	
H12	0.7399	1.0177	0.3144	0.016*	
C13	0.8147 (2)	1.09278 (13)	0.1744 (2)	0.0152 (4)	
C14	0.7829 (2)	1.14153 (14)	0.0594 (2)	0.0172 (4)	
H14	0.8558	1.1685	0.0195	0.021*	
C15	0.6456 (2)	1.15045 (14)	0.00394 (19)	0.0158 (4)	
H15	0.6215	1.1845	−0.0728	0.019*	
C16	0.5443 (2)	1.10761 (13)	0.06493 (19)	0.0138 (4)	

### Atomic displacement parameters (Å^2^)

**Table:**

	*U*^11^	*U*^22^	*U*^33^	*U*^12^	*U*^13^	*U*^23^
Br1	0.01187 (12)	0.03131 (14)	0.02403 (13)	−0.00169 (9)	0.00198 (8)	0.00285 (9)
O1	0.0119 (7)	0.0181 (7)	0.0178 (7)	−0.0008 (6)	0.0047 (6)	0.0012 (6)
O2	0.0100 (7)	0.0235 (8)	0.0151 (7)	−0.0005 (6)	0.0010 (6)	0.0048 (6)
O3	0.0120 (7)	0.0162 (7)	0.0122 (6)	0.0003 (6)	0.0039 (5)	0.0011 (5)
O4	0.0101 (7)	0.0174 (7)	0.0145 (7)	−0.0035 (6)	0.0005 (5)	0.0038 (5)
N1	0.0114 (9)	0.0144 (8)	0.0152 (8)	−0.0019 (7)	0.0050 (7)	0.0021 (7)
N2	0.0149 (9)	0.0123 (8)	0.0125 (7)	−0.0020 (7)	0.0051 (7)	−0.0006 (6)
N3	0.0158 (9)	0.0156 (9)	0.0133 (8)	0.0028 (7)	−0.0004 (7)	−0.0001 (6)
C1	0.0123 (10)	0.0122 (9)	0.0133 (9)	−0.0012 (8)	0.0044 (7)	−0.0035 (7)
C2	0.0093 (9)	0.0150 (9)	0.0150 (9)	0.0001 (8)	0.0031 (7)	−0.0024 (8)
C3	0.0116 (10)	0.0149 (10)	0.0116 (8)	0.0016 (8)	−0.0002 (7)	−0.0027 (7)
C4	0.0133 (10)	0.0111 (9)	0.0109 (8)	−0.0017 (7)	0.0035 (7)	−0.0017 (7)
C5	0.0101 (9)	0.0126 (9)	0.0143 (9)	−0.0010 (8)	0.0025 (7)	−0.0042 (7)
C6	0.0119 (10)	0.0124 (9)	0.0127 (9)	0.0006 (8)	0.0009 (7)	0.0003 (7)
C7	0.0148 (10)	0.0114 (9)	0.0131 (9)	−0.0005 (8)	0.0046 (8)	−0.0044 (7)
C8	0.0117 (10)	0.0133 (9)	0.0155 (9)	−0.0012 (8)	0.0042 (7)	−0.0031 (8)
C9	0.0131 (10)	0.0104 (9)	0.0141 (9)	0.0005 (8)	0.0018 (8)	−0.0029 (7)
C10	0.0144 (10)	0.0146 (9)	0.0162 (9)	0.0008 (8)	0.0027 (8)	−0.0021 (8)
C11	0.0148 (10)	0.0099 (9)	0.0119 (8)	0.0009 (8)	0.0032 (7)	−0.0025 (7)
C12	0.0165 (10)	0.0125 (9)	0.0116 (9)	0.0023 (8)	0.0028 (7)	−0.0013 (7)
C13	0.0132 (10)	0.0146 (10)	0.0176 (9)	0.0000 (8)	0.0017 (8)	−0.0028 (8)
C14	0.0194 (11)	0.0149 (10)	0.0185 (10)	−0.0026 (9)	0.0072 (8)	−0.0002 (8)
C15	0.0233 (11)	0.0116 (9)	0.0128 (9)	0.0002 (8)	0.0035 (8)	0.0004 (7)
C16	0.0166 (10)	0.0117 (9)	0.0126 (9)	0.0021 (8)	0.0005 (8)	−0.0024 (7)

### Geometric parameters (Å, °)

**Table:**

Br1—C13	1.899 (2)	C2—H2	0.9500
O1—C7	1.236 (3)	C3—C4	1.392 (3)
O2—C3	1.375 (2)	C4—C5	1.391 (3)
O2—H2O	0.8400	C5—C6	1.387 (3)
O3—C4	1.368 (2)	C6—H6	0.9500
O3—H3O	0.8400	C8—C9	1.432 (3)
O4—C5	1.365 (2)	C8—H8	0.9500
O4—H4O	0.8400	C9—C10	1.381 (3)
N1—C7	1.346 (3)	C9—C11	1.443 (3)
N1—N2	1.394 (2)	C10—H10	0.9500
N1—H1N	0.8800	C11—C12	1.401 (3)
N2—C8	1.290 (3)	C11—C16	1.406 (3)
N3—C10	1.354 (3)	C12—C13	1.379 (3)
N3—C16	1.378 (3)	C12—H12	0.9500
N3—H3N	0.8800	C13—C14	1.400 (3)
C1—C6	1.388 (3)	C14—C15	1.381 (3)
C1—C2	1.398 (3)	C14—H14	0.9500
C1—C7	1.497 (3)	C15—C16	1.388 (3)
C2—C3	1.382 (3)	C15—H15	0.9500
			
C3—O2—H2O	109.5	O1—C7—C1	120.77 (18)
C4—O3—H3O	109.5	N1—C7—C1	116.61 (18)
C5—O4—H4O	109.5	N2—C8—C9	122.40 (19)
C7—N1—N2	119.79 (17)	N2—C8—H8	118.8
C7—N1—H1N	120.1	C9—C8—H8	118.8
N2—N1—H1N	120.1	C10—C9—C8	123.76 (19)
C8—N2—N1	114.89 (17)	C10—C9—C11	106.28 (17)
C10—N3—C16	109.48 (17)	C8—C9—C11	129.92 (19)
C10—N3—H3N	125.3	N3—C10—C9	109.93 (19)
C16—N3—H3N	125.3	N3—C10—H10	125.0
C6—C1—C2	120.15 (18)	C9—C10—H10	125.0
C6—C1—C7	122.57 (18)	C12—C11—C16	119.25 (18)
C2—C1—C7	117.28 (18)	C12—C11—C9	134.17 (18)
C3—C2—C1	118.93 (18)	C16—C11—C9	106.54 (18)
C3—C2—H2	120.5	C13—C12—C11	117.12 (18)
C1—C2—H2	120.5	C13—C12—H12	121.4
O2—C3—C2	120.06 (18)	C11—C12—H12	121.4
O2—C3—C4	118.49 (17)	C12—C13—C14	123.3 (2)
C2—C3—C4	121.44 (18)	C12—C13—Br1	118.96 (15)
O3—C4—C3	117.54 (18)	C14—C13—Br1	117.63 (16)
O3—C4—C5	123.37 (18)	C15—C14—C13	119.95 (19)
C3—C4—C5	119.04 (18)	C15—C14—H14	120.0
O4—C5—C6	117.06 (17)	C13—C14—H14	120.0
O4—C5—C4	122.77 (17)	C14—C15—C16	117.31 (19)
C6—C5—C4	120.17 (19)	C14—C15—H15	121.3
C1—C6—C5	120.23 (18)	C16—C15—H15	121.3
C1—C6—H6	119.9	N3—C16—C15	129.25 (19)
C5—C6—H6	119.9	N3—C16—C11	107.77 (18)
O1—C7—N1	122.61 (18)	C15—C16—C11	122.99 (19)
			
C7—N1—N2—C8	−175.49 (18)	N2—C8—C9—C10	166.76 (19)
C6—C1—C2—C3	−1.3 (3)	N2—C8—C9—C11	−10.6 (3)
C7—C1—C2—C3	178.65 (17)	C16—N3—C10—C9	0.0 (2)
C1—C2—C3—O2	−179.39 (17)	C8—C9—C10—N3	−177.81 (18)
C1—C2—C3—C4	−0.4 (3)	C11—C9—C10—N3	0.1 (2)
O2—C3—C4—O3	−1.8 (3)	C10—C9—C11—C12	177.6 (2)
C2—C3—C4—O3	179.23 (17)	C8—C9—C11—C12	−4.7 (4)
O2—C3—C4—C5	−179.37 (17)	C10—C9—C11—C16	−0.1 (2)
C2—C3—C4—C5	1.7 (3)	C8—C9—C11—C16	177.60 (19)
O3—C4—C5—O4	0.9 (3)	C16—C11—C12—C13	−1.9 (3)
C3—C4—C5—O4	178.29 (17)	C9—C11—C12—C13	−179.3 (2)
O3—C4—C5—C6	−178.58 (18)	C11—C12—C13—C14	0.2 (3)
C3—C4—C5—C6	−1.2 (3)	C11—C12—C13—Br1	177.22 (14)
C2—C1—C6—C5	1.8 (3)	C12—C13—C14—C15	1.6 (3)
C7—C1—C6—C5	−178.16 (18)	Br1—C13—C14—C15	−175.44 (15)
O4—C5—C6—C1	179.97 (17)	C13—C14—C15—C16	−1.7 (3)
C4—C5—C6—C1	−0.6 (3)	C10—N3—C16—C15	−179.9 (2)
N2—N1—C7—O1	2.9 (3)	C10—N3—C16—C11	0.0 (2)
N2—N1—C7—C1	−176.32 (16)	C14—C15—C16—N3	179.8 (2)
C6—C1—C7—O1	−174.49 (18)	C14—C15—C16—C11	0.0 (3)
C2—C1—C7—O1	5.5 (3)	C12—C11—C16—N3	−178.04 (17)
C6—C1—C7—N1	4.7 (3)	C9—C11—C16—N3	0.1 (2)
C2—C1—C7—N1	−175.23 (17)	C12—C11—C16—C15	1.8 (3)
N1—N2—C8—C9	−178.50 (17)	C9—C11—C16—C15	179.91 (18)

### Hydrogen-bond geometry (Å, °)

**Table:**

*D*—H···*A*	*D*—H	H···*A*	*D*···*A*	*D*—H···*A*
O2—H2O···O3	0.84	2.21	2.681 (2)	116
O3—H3O···O1^i^	0.84	1.76	2.595 (2)	173
O4—H4O···N2^i^	0.84	2.02	2.778 (2)	150
N1—H1N···O2^ii^	0.88	2.26	3.111 (2)	163
N3—H3N···O4^iii^	0.88	2.11	2.932 (2)	154

Symmetry codes: (i) *x*−1/2, −*y*+3/2, *z*+1/2; (ii) *x*−1/2, −*y*+3/2, *z*−1/2; (iii) −*x*+1/2, *y*+1/2, −*z*+1/2.
